# Analysis of long-term family dynamics in mothers who have undergone fetal myelomeningocele surgery using telemedicine: a pilot study

**DOI:** 10.1590/1806-9282.20231327

**Published:** 2024-06-17

**Authors:** Tatiane Santos Nunes, Edward Araujo, Liliam Cristine Rolo, Adriana Sañudo, Sergio Cavalheiro, Antonio Fernandes Moron

**Affiliations:** 1 Universidade Federal de São Paulo, Escola Paulista de Medicina, Department of Obstetrics - São Paulo (SP), Brazil.; 2 Universidade Federal de São Paulo, Escola Paulista de Medicina, Department of Preventive Medicine - São Paulo (SP), Brazil.; 3 Universidade Federal de São Paulo, Escola Paulista de Medicina, Department of Neurology and Neurosurgery - São Paulo (SP), Brazil.

**Keywords:** Spina bifida, Surgery, Follow-up study, Family dynamics, Telemedicine

## Abstract

**OBJECTIVE:**

The aim of this study was to understand the dynamics of families with children with myelomeningocele undergoing intrauterine fetal surgery.

**METHODS:**

A retrospective cohort pilot study was carried out with 11 mothers of children who had undergone intrauterine myelomeningocele repair. Participants in this study responded to an electronic questionnaire (via Google Forms), developed by the study authors, that consisted of 22 multiple-choice questions, of which 17 were closed-ended and 5 had a standardized format.

**RESULTS:**

The mean (± standard deviation) of the mothers’ age was 37.6 (± 3.5) years. The median of gestational age at delivery and birthweight were 34.9 (range, 33 to 36.1) weeks and 2,300 (range, 1,950 to 2,763) g, respectively. The majority of mothers were white (81.8%), had university degree (81.8%), were Catholic (63.6%), and were married (100%). The majority of mothers rated their relationship with their husband, family, and friends as excellent (54.5, 72.7, and 54.5%, respectively). All 11 mothers reported that the newborn with myelomeningocele was born <37 weeks gestation and the birthweight most often<2,500 g. Approximately 64% of the mothers reported that their child required adaptations or had special needs, of which walking aids (50%) and bladder control (50%) were the most common ones.

**CONCLUSION:**

Telemedicine proved to be a useful tool in the long-term follow-up of children who underwent intrauterine surgery to correct myelomeningocele.

## INTRODUCTION

Fetal myelomeningocele is considered a severe form of neural tube closure disorder, resulting from a failure of the primary neurulation process, with closure normally occurring in the fourth week of gestation^1^. It affects approximately 2,000 live births worldwide^2^, with an incidence of approximately 3.4—6 per 10,000 newborns in the United States^3^. The main clinical alterations of myelomeningocele are motor and cognitive deficits, hydrocephalus, varying degrees of skeletal delay and motor deformities, and bladder and bowel dysfunction throughout life, requiring prolonged assistance from the child's caregiver to perform basic activities of daily living^4^.

Myelomeningocele is one of the most serious congenital defects and is considered a nonlethal fetal anomaly for which there is no satisfactory postnatal treatment, with significant morbidities, including dysfunction of the bowel, bladder, and reproductive organs, requiring a careful surgical procedure and careful analysis of risks and benefits^5^. Studies have shown improved quality of life for fetuses subjected to intrauterine surgery, minimized exposure of neural elements to amniotic fluid, improved cerebellar herniation, reduced hydrocephalus, as well as a significant contribution to prognosis and reduced economic costs of treating the disease in the postnatal period^6^. Other benefits of intrauterine surgery include a reduction in ventriculo-peritoneal shunt rates by 12 months of age and the ability to walk by 30 months of age^7^.

Currently, most developed countries offer telehealth services. In developing countries, on the other hand, telemedicine faces a major health challenge in terms of expanding access to specialized medical services in places where the quality of health care has not improved^8^. It should be noted that the care of a child with myelomeningocele does not end in the postnatal period, but throughout life, this care will be continuous, actively requiring the involvement of the family. In this scenario, the use of the telemedicine tool becomes indispensable in accompanying families, so that it can help them care for their child who will need special care and teams trained to accompany them^9^. In addition, the multi-professional team should know the dynamics of each family, taking into account its individuality in order to recognize its different needs, personalize the practice of care, develop strategies that promote the child's autonomy, preserve its cognitive functions, and promote an improved quality of life^10^.

Therefore, the aim of the present study was to identify the family dynamics of children diagnosed with fetal myelomeningocele who underwent intrauterine corrective surgery using the telemedicine tool.

## METHODS

A retrospective cohort pilot study was carried out between 2018 and 2023 with 11 mothers of children who had undergone intrauterine myelomeningocele repair. This study was approved by the Ethics Committee of the Federal University of São Paulo (UNIFESP) and the mothers signed a consent form. To participate in the study, mothers received an invitation letter via email and WhatsApp. Patients with severe cognitive impairment and those who refused to participate at any point in the study were excluded.

Participants in this study responded to an electronic questionnaire (via Google Forms), developed by the study authors, that consisted of 22 multiple-choice questions, of which 17 were closed-ended and 5 had a standardized format. The first part of the questionnaire was designed to assess the socio-demographic profile of the participants and, through closed-ended questions, to understand the ongoing family and psychological impact of the diagnosis of myelomeningocele and its impact on pregnancy. The second part was designed to understand the family dynamics after the intrauterine correction surgery. The estimated time to complete the questionnaire was approximately 25 min, but as much time as necessary could be used. The questionnaire could not be completed until the last question had been answered.

To gain a better understanding of daily routines and family dynamics, a telemedicine interview was scheduled, with a time and date based on the participant's availability. On the agreed day and time, a telemedicine consultation was scheduled through a secure commercial platform (Versatilis^Ò^), and the participant received an electronic access link.

In the present study, telemedicine was used as a tool to facilitate virtual interviews and complement the research, allowing the researchers (nurse and physician) to compare the participants’ responses and the level of satisfaction expressed by the families. In addition, participants were able to share their experiences through the interactive audiovisual communication method offered by the telemedicine tool, which was crucial given the expertise and guidance provided by health professionals. Another factor observed by the researchers was the convenience and flexibility of choosing the best day and time for the interview, regardless of the distance, as participants expressed satisfaction, affection, and confidence during the interview.

The data was transferred to an Excel 2010 spreadsheet (Microsoft Corp., Redmond, WA, USA) and analyzed using the STATA/SE 15.1 (Stata Corp., College Station, TX, USA) statistical program. Qualitative variables were described as number (N) and percentage (%), while quantitative variables were described as mean (standard deviation—SD), minimum and maximum values, or as median, 25th and 75th percentile, and minimum and maximum values.

## RESULTS

We selected 13 mothers, but 2 refused to participate in the study, leaving 11 mothers in the final sample. All of them completed the questionnaire in full, with a 100% success rate. Only 2 (18.2%) of the mothers interviewed had tried to get pregnant again, and most of them reported fear and insecurity about being diagnosed with fetal myelomeningocele again. The mean (± SD) mothers’ age was 37.6 (± 3.5) years. The majority of mothers were white (81.8%), had university degree (81.8%), were Catholic (63.6%), and were married (100%). The majority of mothers rated their relationship with their husband, family, and friends as excellent (54.5, 72.7, and 54.5%, respectively). Of the six mothers who have other children, about 83% rated their relationship with them as excellent. Table 1 shows the description of the socioeconomic and demographic data as well as the relationships with their husband, children, and friends.

**Table 1:** Description of socioeconomic and demographic data and relationship with husband/children/friends.

Variables		N (%)
Age, years		
	Mean (standard deviation)	37.6 (3.5)
	Minimum–maximum	32–44
Gestational age at delivery, weeks		
	Median	34.9
	Minimum–maximum	33–36.1
Birthweight, g		
	Median	2,300
	Minimum–maximum	1,950–2,763
Race		
	White	9 (81.8%)
	No-white	2 (18.2%)
Schooling		
	Incomplete university degree	2 (18.2%)
	University degree	9 (81.8%)
Religion		
	Catholic	7 (63.6%)
	Spiritist	2 (18.2%)
	Evangelica	1 (9.1%)
	Marital status, married	11 (100%)
	Lives with partner, yes	11 (100%)
Relationship with the partner		
	Great	6 (54.5%)
	Good	2 (18.2%)
	Regular	2 (18.2%)
	Not applicable	1 (9.1%)
Relationship with the family		
	Great	8 (72.7%)
	Good	2 (18.2%)
	Regular	1 (9.1%)
Do you have other children?		
	No	5 (45.5%)
	Yes	6 (54.5%)
Relationship with other children		(n = 6)
	Great	5 (83.3%)
	Good	1 (16.7%)
Relationship with friends		
	Great	6 (54.5%)
	Good	5 (45.5%)

Table 2 showed that most of the mothers reported 2 (45.4%) previous pregnancies, the most frequent number of deliveries was 1 (45.4%), approximately 91% of the women reported not having had abortions, and all of them reported no stillbirths or neonatal deaths. As for the number of children, the most frequent answer was 1 (45.4%) child. When asked about the number of preterm births, 9 (81.8%) of them reported 1. All 11 mothers reported that the newborn with myelomeningocele was delivered with a median of 34.9 (range, 33 to 36.1) weeks’ gestation and a birthweight median of 2,300 (range, 1,950 to 2,763) g. Only one woman reported a history of fetal malformation, which was a neural tube malformation. Notably, 2 (18.2%) mothers reported a family history of fetal malformations, and 1 of them was myelomeningocele. Only two mothers reported that they intended to get pregnant again. Most of the mothers (n = 7, 63.6%) had undergone previous surgical procedures, and the most frequent procedure was intrauterine surgery for myelomeningocele (n = 4, 57.1%).

**Table 2:** Maternal history and perinatal outcomes.

Variables		N (%)
Number of previous pregnancies		
	1	4 (36.4%)
	2	5 (45.4%)
	3	2 (18.2%)
Number of deliveries		
	1	5 (45.4%)
	2	4 (36.4%)
	3	2 (18.2%)
Number of abortions		
	None	10 (90.9%)
	1	1 (9.1%)
Number of live children		
	1	5 (45.4%)
	2	4 (36.4%)
	3	2 (18.2%)
	Stillbirth, none	11 (100%)
	Stillborn, none	11 (100%)
Number of preterm births (<37 weeks)		
	1	9 (81.8%)
	2	2 (18.2%)
Number of post-term births (≥42 weeks)		
	0	10 (90.9%)
	1	1 (9.1%)
Birthweight of newborn with myelomeningocele		
	<2,500 g	8 (72.7%)
	2,500–4,000 g	3 (27.3%)
Gestational age at delivery of newborn with myelomeningocele		
	<37 weeks	11 (100%)
History of fetal malformation		
	No	10 (90.9%)
	Yes	1 (9.1%)
What? Neural tube defect		
Family history of fetal malformation		
	No	9 (81,8%)
	Yes	2 (18,2%)
What?		
	No definitive diagnosis	1 (50%)
	Myelomeningocele	1 (50%)
Do you intend to get pregnant again?		
	No	9 (81.8%)
	Yes	2 (18.2%)
If you want to get pregnant, do you plan it?		
	Yes	2 (100%)

Table 3 shows the description of the activities currently carried out with the child, being that all the children have some kind of follow-up with a specialist, with physiotherapy and neurology the most frequently reported (91%). Approximately 64% of the mothers reported that their child required adaptations or had special needs. Most of the children did not use wheelchairs, walkers, or crutches (81.8 and 72.7%, respectively). However, the majority of children, approximately 73%, used orthopedic shoes or special orthoses. Regarding the need for adaptations, the most common response was that there was no need for adaptations, both at home and in the car (72.7 and 90.9%, respectively). More than 70% of the children required some type of daily procedure, the most common being the use of disposable diapers (75%) and intermittent catheterization (50%). The special needs most often reported by mothers were walking aids (50%) and bladder control (50%).

**Table 3:** Description of current activities with your child.

Variables		N (%)
Do you have any kind of specialist follow-up?		
	Yes	11 (100%)
Type of follow-up		(n = 11)
	Physiotherapy	10 (90.9%)
	Neurology	10 (90.9%)
	Urology	9 (81.8%)
	Orthopedics	9 (81.8%)
	Occupational therapy	4 (36.4%)
	Psychology	3 (27.3%)
	Speech therapy	2 (18.2%)
	Orthodontists	1 (9.1%)
	Other specialists	4 (36.4%)
How often?		
	One a weak	1 (9.1%)
	Three times a weak	2 (18.2%)
	Others	8 (72.7%)
Does your child need adaptations or special needs?		
	No	4 (36.4%)
	Yes	7 (63.6%)
Use of a wheelchair		
	No	9 (81.8%)
	Yes	2 (18.2%)
Use of a walker or crutches		
	No	8 (72.7%)
	Yes	3 (27.3%)
Orthopedic shoes or special orthoses		
	No	3 (27.3%)
	Yes	8 (72.7%)
Have you had to make any changes or adaptations to your home to better accommodate your child?		
	No	8 (72.7%)
	Yes	3 (27.3%)
Changing or adapting your car		
	No	10 (90.9%)
	Yes	1 (9.1%)
Does your child need any daily procedures?		
	No	3 (27.3%)
	Yes	8 (72.7%)
What are the daily procedures?		(n = 8)
	Use of disposable diapers	6 (75%)
	Intermittent bladder catheterization	4 (50%)
	Other products/materials	1 (12.5%)
Special needs		(n = 8)
	Walking aid	4 (50%)
	Fecal elimination	4 (50%)
	Bladder elimination	3 (37.5%)
	Supporting oneself	2 (25%)
	Feeding	1 (12.5%)
	Speech difficulties	1 (12.5%)
	Holding an object	1 (12.5%)
	Difficulty interacting with other children	1 (12.5%)

## DISCUSSION

The active participation of the family is essential for the child's development and autonomy. Parents, for their part, try to learn how to adapt their routine and meet their child's needs in order to overcome the greatest challenges and become as independent as possible, especially in the adult phase. From this perspective, it is necessary for the professional responsible for the rehabilitation of children with myelomeningocele to know the dynamics of each family and to develop strategies for personalized care, especially those related to family-centered care and not just that of the child^10^.

After intrauterine surgery for myelomeningocele, women are advised not to become pregnant for the first 2 years. Of the mothers, 2 (18.8%) did not try to become pregnant because they feared complications in subsequent pregnancies. Goodnight et al.^11^ assessed the obstetric risk in subsequent pregnancies after intrauterine repair of myelomeningocele. From 693 cases of intrauterine surgeries, 77 subsequent pregnancies in 60 women were observed. The uterine rupture rate was 9.6% (n = 5), resulting in 2 fetal deaths, and maternal transfusion was required in 4 (7.7%) patients.

In our study, 7 (63.6%) of the children required special care, including 8 (72.7%) with orthopedic shoes or special orthoses. In a pioneer study developed in our service after the first six intrauterine repairs for myelomeningocele at the age of 3.5 years, two children had normal leg movements, sacral functional level, and were community ambulators. One child with a lesion at the L1–L2 anatomical level was nonambulatory and completely dependent on a wheelchair for mobility^12^.

This study showed that most of the children have special needs, but there was no need to adapt the family home or car. Buoro and Nogueira^13^ searched to identify the main challenges facing the family of a child with meningomyelocele. They observed that the quality of life of mothers and caregivers of children with meningomyelocele was affected regarding functional capacity, emotional aspects, and mental health. The most common difficulties faced by caregivers were performing bladder catheterization, providing general care, financial burden, and accessibility.

Intrauterine surgery for myelomeningocele is associated with a higher risk of adverse outcomes for the maternal–fetal binomial. In our study, all deliveries were <37 weeks, and the birthweight<2,500 g occurred in 8 (72.3%) cases. Moron et al.^14^ assessed the perinatal outcomes of 237 intrauterine surgeries for myelomeningocele. The mean gestational age at delivery and birthweight were 33.6 ± 2.4 weeks and 2,186 ± 506 g, respectively, and 86.9% of deliveries were <37 weeks.

A comparative multicenter study assessed the long-term impact on families and caregivers of children with myelomeningocele who underwent intrauterine surgery in the pre- and postnatal periods. They found that the group who underwent intrauterine surgery had a lower social impact on the family when compared to the group who underwent postnatal surgical correction^15^.

This study showed that the active participation of the family was essential for the development and autonomy of the children. Parents try to learn the necessary care to adapt to the routine and meet the needs of their children so that they can overcome their major challenges and become increasingly independent. In this scenario, professionals working to rehabilitate these children must know the dynamics of each family and develop personalized care strategies that focus on the families, not just the children.

Some studies describe the benefits and ease of use of telemedicine in health services, highlighting the use of technology for interventions in maternal and child health. The main objective is to improve not only care, but also prenatal care, postnatal follow-up, and child care, and to reduce the risk of maternal and neonatal mortality or morbidity^16,17^. In Arkansas, telemedicine is commonly used in obstetrics through the Antenatal and Neonatal Guidelines, Education and Learning System (ANGELS), the statewide telemedicine network. This network is primarily used for teleultrasound consultations and maternal–fetal medicine^18^.

Obviously, the frequency of telemedicine encounters has increased significantly in recent years. We can still emphasize the safety and quality of the services provided by this tool in the field of obstetrics and fetal medicine. These include prenatal care, postpartum care, diabetes mellitus management, medication abortion, lactation support, hypertension control, genetic counseling, ultrasound, contraception, and mental health services. For many users of these services, telemedicine has several potential or proven benefits, including expanded patient access, increased patient satisfaction, and reduced disparities in care and health outcomes compared to in-person encounters^19^.

In the current study, most of the participants used the telemedicine tool successfully, showing trust and satisfaction to the researchers, i.e., the remote format did not impede the effectiveness of the care. The knowledge generated by this study will contribute to the support of different educational and professional actions in the care of pregnant women, from the prenatal period to the postnatal period. Such knowledge will strengthen the professional–patient bond, with benefits such as improving access to postnatal care, promoting better integration, and improving clinical outcomes, even for those who live in places with difficult access. In addition, the new knowledge will provide continuity of care, bringing patients closer to the professional and allowing them 'autonomy' and security. We believe that the results of this study will contribute to the expansion of primary care, extending to health reference services, especially those of high complexity and high-risk pregnancies, as well as those of fetal medicine throughout the country.

## CONCLUSION

Telemedicine proved to be a useful tool in the long-term follow-up of children who underwent intrauterine surgery to correct myelomeningocele. The majority of children required special care, with walking aids and bladder control being the most common. Also, this study showed that the use of technological tools in monitoring families is fundamental and serves as a strategic support model. We emphasize that the use of these tools in long-term monitoring can provide a better quality of life for children and support for their families, strengthening the bond and promoting a closer relationship between professionals and their clients/patients.
